# Functional disruption of human leukocyte antigen II in human embryonic stem cell

**DOI:** 10.1186/s40659-015-0051-6

**Published:** 2015-10-27

**Authors:** Haide Chen, Yang Li, Xijuan Lin, Di Cui, Chun Cui, Hui Li, Lei Xiao

**Affiliations:** College of Animal Science, Zhejiang University, Hangzhou, 310058 People’s Republic of China; Zhejiang University School of Medicine, Hangzhou, 310058 People’s Republic of China; Wuxi Medical School, Jiangnan University, Wuxi, 214122 People’s Republic of China; Xiangtan Center Hospital, Hunan, 411100 People’s Republic of China

**Keywords:** hESCs, *CIITA*, TALENs, Immune rejection

## Abstract

**Background:**

Theoretically human embryonic stem cells (hESCs) have the capacity to self-renew and differentiate into all human cell types. Therefore, the greatest promise of hESCs-based therapy is to replace the damaged tissues of patients suffering from traumatic or degenerative diseases by the exact same type of cells derived from hESCs. Allograft immune rejection is one of the obstacles for hESCs-based clinical applications. Human leukocyte antigen (HLA) II leads to CD4^+^ T cells-mediated allograft rejection. Hence, we focus on optimizing hESCs for clinic application through gene modification.

**Results:**

Transcription activator-like effector nucleases (TALENs) were used to target MHC class II transactivator (*CIITA*) in hESCs efficiently. *CIITA*^−/−^ hESCs did not show any difference in the differentiation potential and self-renewal capacity. Dendritic cells (DCs) derived from *CIITA*^−/−^ hESCs expressed CD83 and CD86 but without the constitutive HLA II. Fibroblasts derived from *CIITA*^−/−^ hESCs were powerless in IFN-γ inducible expression of HLA II.

**Conclusion:**

We generated HLA II defected hESCs via deleting *CIITA*, a master regulator of constitutive and IFN-γ inducible expression of HLA II genes. *CIITA*^−/−^ hESCs can differentiate into tissue cells with non-HLA II expression. It’s promising that *CIITA*^−/−^ hESCs-derived cells could be used in cell therapy (e.g., T cells and DCs) and escape the attack of receptors’ CD4^+^ T cells, which are the main effector cells of cellular immunity in allograft.

**Electronic supplementary material:**

The online version of this article (doi:10.1186/s40659-015-0051-6) contains supplementary material, which is available to authorized users.

## Background

Since the first establishment of hESCs by Thomson’s group in 1998 [1], many therapy strategies based on hESCs have been attempted to cure human diseases. Nevertheless, several major obstacles remain to be addressed before clinical applications of hESCs-based cells replacement therapy, such as allograft immune rejection. Hence, we focus on generating hypoimmunogenic and universally compatible hESCs for clinical use, which can attenuate the effect of T cell-mediated rejection.

The activation of T cells is based on two signals (TCR-HLA signal and costimulatory signal). HLA molecules are encoded by a large gene family and divided into class I and II. Firstly, professional or non-professional antigen-presenting cells (APCs) degrade proteins into peptides and then load these peptides onto HLA molecules. And then, TCRs of CD4^+^ and CD8^+^ T cells recognized the peptides presented by HLA II and HLA I, respectively. At the same time, those APCs must express a spectrum of costimulatory molecules (e.g., CD80 and CD86), which will interact with complementary molecules of T cells (e.g., CD28 and Cytotoxic T lymphocyte antigen 4 (CTLA4)). Both TCR-HLA signal and costimulatory signal are required for activation of T cells [2]. Thus, if we inhibit either of them, T cells would not attack the allografts. We chose to delete HLA molecules. It has been proved that hESCs expressing CTLA4-immunoglobulin fusion protein (CTLA4-Ig) and programmed death ligand-1 (PD-L1) can suppress the allogeneic immune response by simultaneously disrupting the costimulatory pathway and activating the T cell inhibitory pathway [3, 4]. This strategy is useful but not generally applicable. For example, T cells derived from hESCs can’t be activated with the expression of CTLA4-Ig and PD-L1. So it will limit the application of hESCs in clinic immunotherapy, such as hESCs-derived chimeric antigen receptor (CAR)-T, an effective therapy in cancer treatment [5]. Moreover, unlike mice T cells, activated human T cells will express HLA II. So our strategy has an advantage to produce hypoimmunogenic and universally compatible CAR-T, and they can prevent the rejection mediated by recipients’ T cells. Furthermore, we can also derive DCs from those hESCs without HLA II. Though those DCs can’t present antigens normally, the CAR technique (CAR-DCs) [6] and artificial HLA-peptide [7, 8] will let them be more specific and sensitive to the cancers.

HLA I molecules are found on the surface of each nucleated cells [9]. Constitutive HLA II molecules are expressed mainly on thymic epithelial cells and professional APCs, including DCs, B-lymphocytes, monocytes and macrophages. Under the stress of inflammatory cytokines (e.g., IFN-γ and TNF-α), nonprofessional APCs such as fibroblasts and epithelial cells can also express HLA II molecules, which are known as “induced HLA II” [10]. Each classical HLA I molecule structurally consists of a polymorphic heavy chain (e.g., HLA-A, HLA-B and HLA-C), which binds to a same light chain *β2M.* Other group and our lab have knocked out *β2M* in hESCs and demonstrated the loss of HLA I molecules, which endowed hESCs with the capacity of avoiding the CD8^+^ T cells-mediated rejection [11, 12]. Those papers optimized hESCs through HLA I deletion, and this strategy was easy to test because of the constitutive expression of HLA I in each nucleated cells [11–13]. So far, no report has demonstrated the generation of hESCs with the ability of differentiating to cells without constitutive and IFN-γ induced HLA II.

With conserved binding regions, HLA II genes are regulated by a same regulatory complex consists of three RFX factors (RFXAP, RFX5, RFXANK) and CIITA [14, 15]. This complex regulates not only the genes encoding classical HLA II molecules (HLA-DP, HLA-DQ and HLA-DR) but also the genes encoding accessory proteins that are required for intracellular transportation and peptide loading of HLA II molecules, including the non-classical HLA II molecules (invariant chain (Ii), HLA-DM and HLA-DO) [16]. In some cases, tumor cells [17] and virus-infected cells [18, 19] will escape CD4^+^ T cells-mediated immune rejection via silencing the HLA II. Here using TALENs technique, we disrupted HLA II molecules of hESCs by knocking out *CIITA*—the master regulator of HLA II molecules [16, 20]. The main function of *CIITA* is HLA II regulation so they have almost same cellular distribution. *CIITA* does not bind DNA directly but interacts with other elements consisting of cyclic AMP response element-binding protein (CREB), nuclear factor Y complex (NF-Y) and RFX factors (RFX5, RFXANK, RFXAP). Patients without functional *CIITA* are suffering from bare lymphocyte syndrome (BLS), which is characterized by the lack of expression of HLA II in tissue cells [16]. *CIITA*^−/−^ mice are also impaired in MHC class II-mediated allogeneic responses [20].

*CIITA* has four promoters, and they can regulate HLA II expression in a tissue-specific manner [16]. In order to target *CIITA* thoroughly, we designed TALENs in the communal exons (exon 2 and 3) of all transcripts. hESCs don’t express HLA II and *CIITA* in vitro even during the embryoid bodies (EBs) differentiation or IFN-γ induction [21]. We checked the constitutive and induced HLA II molecules on hESCs-derived DCs and fibroblasts, respectively. We found that the deletion of *CIITA* can decrease the constitutive and induced expression of HLA II molecules dramatically.

## Results and discussion

### Disruption of *CIITA* in hESCs by TALENs

**Fig. 1:**
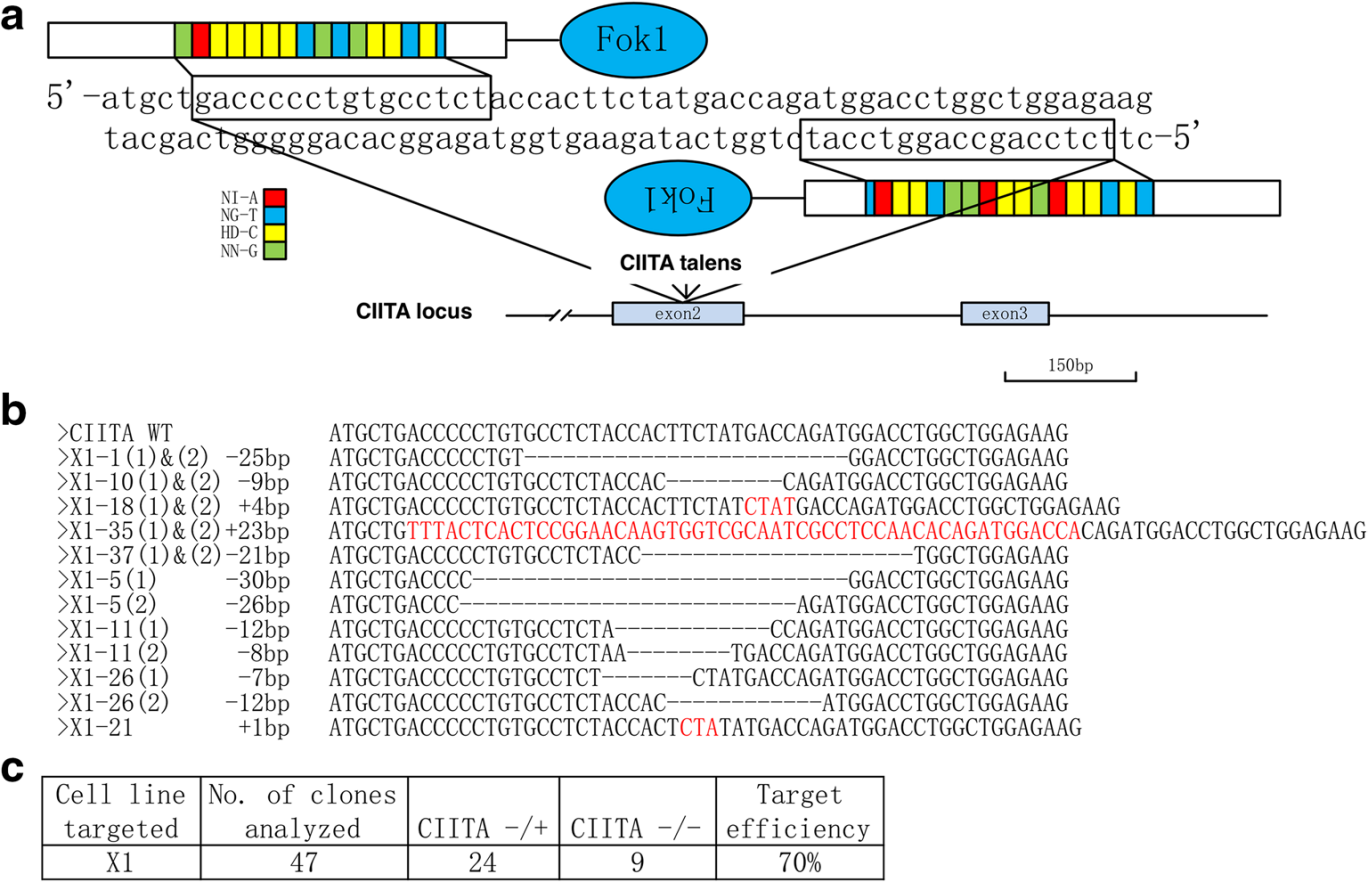
Disruption of *CIITA* in hESCs by TALENs. **a** Sequences on exon 2 of human *CIITA* for target by TALENs.**b** Alignment of the genomic sequences of mutants and wildtypes (wt) at the TALEN target site. The number of deleted (*dashes*) or inserted (*letters in red*) nucleotides compared with the wt sequences is indicated on the *left* of each sequence. **c** Efficiency of generation *CIITA*-deficient hESCs using TALENs in X1

In this study, we targetedly knockout *CIITA* in hESCs with TALENs, which have fewer off-target events than Cas9 and more maneuverable than ZFNs [22]. TALENs of *CIITA* were designed for exon 2 and exon 3 targeting. The most efficient TALEN pairs (2L2 and 2R2) were selected from 293T test and were used to target *CIITA* in X1 hESCs [23] (Fig. 1a). Both heterozygous (*CIITA*^+/−^) and homozygous (*CIITA*^−/−^) hESCs were obtained in one targeting round with the efficiency of 70 % (Fig. 1b, c). Potential off-targets were found by TAL Effector Nucleotide Targeter 2.0 [24] and were checked by sequencing. No mutation was found in potential off-targets (Additional file 1: Table S1).

### The pluripotency of *CIITA* targeted hESCs

**Fig. 2:**
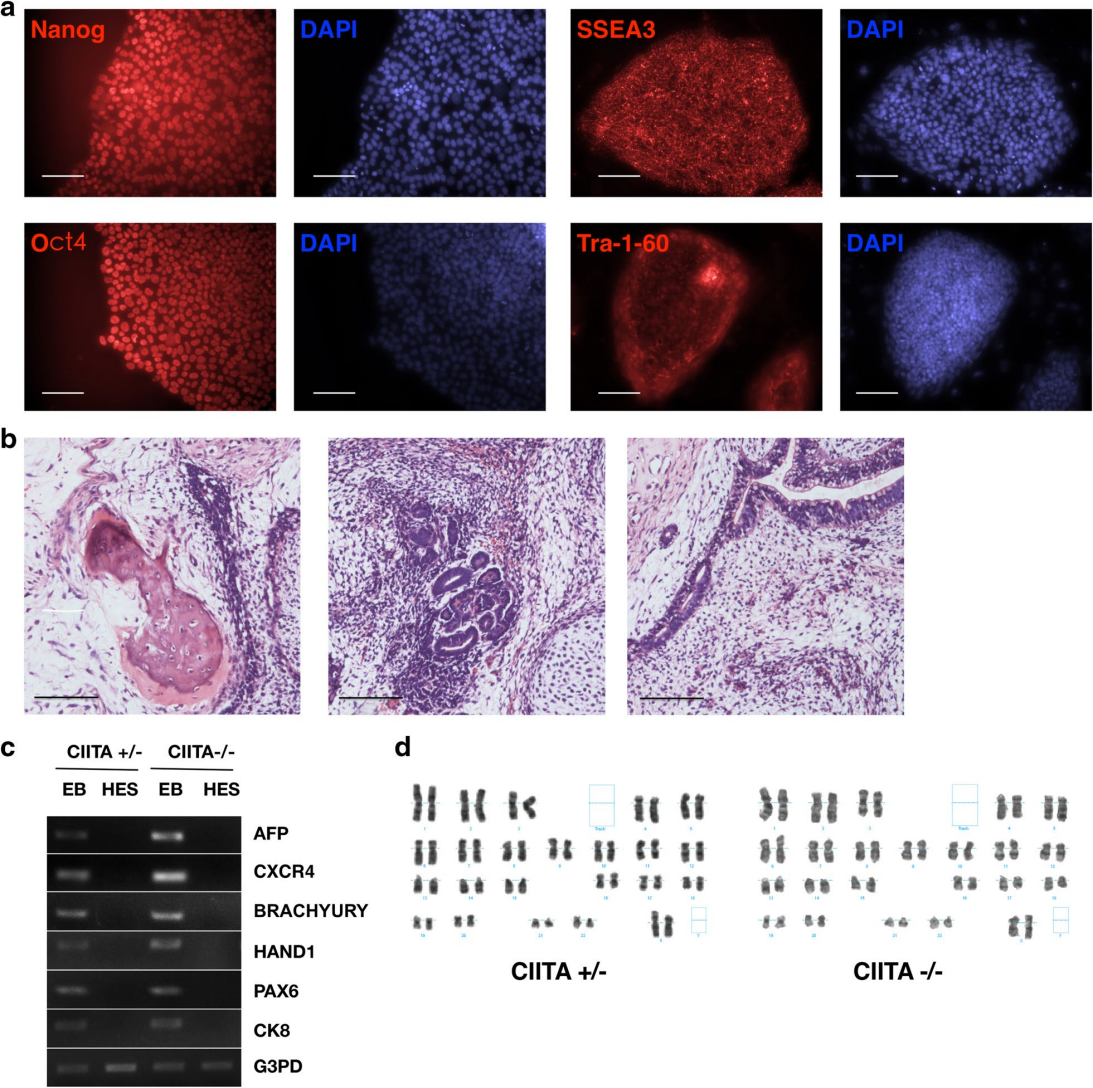
The pluripotency of *CIITA* targeted hESCs. **a** Immunostaining of pluripotent markers, Nanog, Oct4, SSEA3 and Tra-1-60 in *CIITA* ^−/−^ hESCs. **b** HE staining identified three germ layers [mesoderm (*left*), ectoderm (*middle*) and endoderm *(right*)] in teratomas formed from *CIITA* ^−/−^ hESCs. *Scale bar* 100 μm. **c** RT-PCR analysis of differentiated markers expression in *CIITA*-targeted hESC-derived EBs. **d** Karyotype analysis of *CIITA* heterozygous and homozygous hESCs. Both groups had two samples been analyzed and no abnormal karyotype was found

The pluripotency of hESCs are necessary for its application in cells replacement therapy. So we checked the pluripotency in established *CIITA*-targeted hESCs. Immunostaining showed that *CIITA*^−/−^ hESCs were positive for Oct4, Nanog, Tra-1-60 and SSEA3 (Fig. 2a). When *CIITA*^−/−^ hESCs were injected into non-obese diabetic/severe combined immune-deficient (NOD/SCID) mice, teratomas formed after 2 months, and tissues derived from three germ layers were observed in hematoxylin–eosin (HE) staining section (Fig. 2b). EBs derived from *CIITA*^−/−^ hESCs were performed RT-PCR to show the expression of three germ layers markers (Fig. 2c). Moreover, both *CIITA*^−/−^ and *CIITA*^+/−^ hESCs had normal karyotypes (Fig. 2d). We used the normal karyotype lines in the following experiments. Thus the targeted hESCs did not show any difference in pluripotency and karyotypes. So our study would be an attempt to establish clinical hESCs.

### *CIITA* and HLA II expression in defined cells derived from *CIITA* targeted hESCs

Previous reports and our own experiments have pointed out that hESCs don’t express *CIITA* or HLA II, even when they are forming EBs or under IFN-γ induction [21]. Unfortunately, we transplant differentiated cells rather then hESCs into human body directly for the cells replacement therapy. Some tissue cells (e.g., professional APCs and thymic epithelial cells) have constitutive expression of HLA II molecules and some other tissue cells (e.g., fibroblasts and epithelial cells) have induced expression of HLA II molecules. In order to ensure the functional disruption of HLA II, we investigated both kinds of HLA II expression in defined types of cells derived from *CIITA* targeted hESCs.

**Fig. 3:**
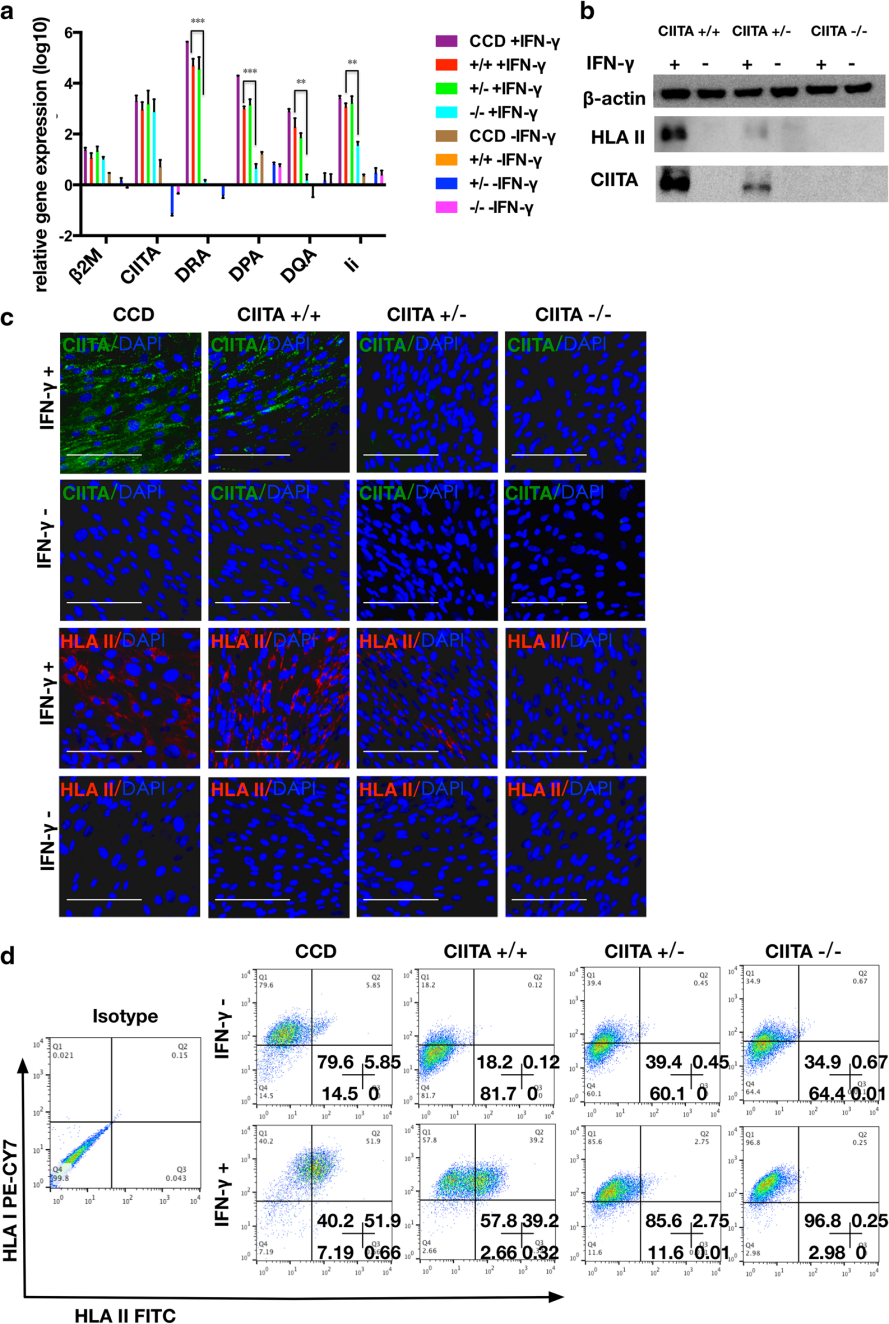
*CIITA* and HLA Class II Expression in fibroblasts derived from *CIITA* targeted hESCs. **a** RT-PCR analysis of *β2M*, *CIITA*, HLA II (*DRA*, *DQA*, *DPA*) and *Ii* in hESCs-derived fibroblasts. They were treated with IFN-γ (500 U/ml) for 5 days. The control groups were IFN-γ free. All groups were compared with *CIITA* ^+/+^ IFN-γfree group. Significance was assessed by a t test. The data are expressed as the mean ± SEM. n ≥ 3. *** p < 0.001, ** P < 0.01. Western blotting (**b**) and immunostaining (**c**) analysis of HLA II and *CIITA* proteins expression in fibroblasts (fibroblasts treated as mentioned above). *Scale bar* 100 μm. **d** FACS analysis of HLA I and II proteins expression on cell surface in fibroblasts (fibroblasts treated as mentioned above)

Firstly, we tested IFN-γ inducible HLA II on hESCs-derived fibroblasts with 5 days’ treatment of 500 U IFN-γ. CCD-1079SK (CCD) cell line, a human fibroblast cell line, was used as a positive control. IFN-γ induction can increase the expression of *β2M* in tissue cells [11]. Without IFN-γ treatment, all cells showed low-level expression of HLA II genes (*CIITA, DRA, DPA, DQA, Ii*) and *β2M*. With IFN-γ treatment, *β2M* and *CIITA* mRNA increased in all groups as reported [11, 16] (Fig. 3a). Our *CIITA* targeting did not affect the transcription of *CIITA* as expected. After IFN-γ treatment, *CIITA*^+/+^ and *CIITA*^+/−^ fibroblasts increased mRNA expression of HLA II genes (*DRA, DPA, DQA, Ii*) as CCD cells did (Fig. 3a). However, IFN-γ treated *CIITA*^−/−^ fibroblasts didn’t increase mRNA expression of HLA II genes (*DRA, DPA, DQA, Ii*) obviously (Fig. 3a). It suggested that *CIITA* mRNA detected in IFN-γ treated *CIITA*^−/−^ fibroblasts was dysfunctional and couldn’t be translated into a functional protein to regulate the expression of HLA II (Fig. 3a). It was proved by the following Western blotting and Immunochemistry data (Fig. 3b, c). It also indicated that *CIITA*^+/−^ fibroblasts had a low level increase of *CIITA* and HLA II protein lagged behind the increase of mRNAs (Fig. 3b, c). FACS analysis of all groups demonstrated that few cells expressed HLA II on cell surface without IFN-γ induction. After IFN-γ induction, CCD and *CIITA*^+/+^ fibroblasts increased expression of HLA I and II dramatically. However, neither *CIITA*^+/−^ nor *CIITA*^−/−^ increased expression of HLA II obviously (Fig. 3d).

**Fig. 4:**
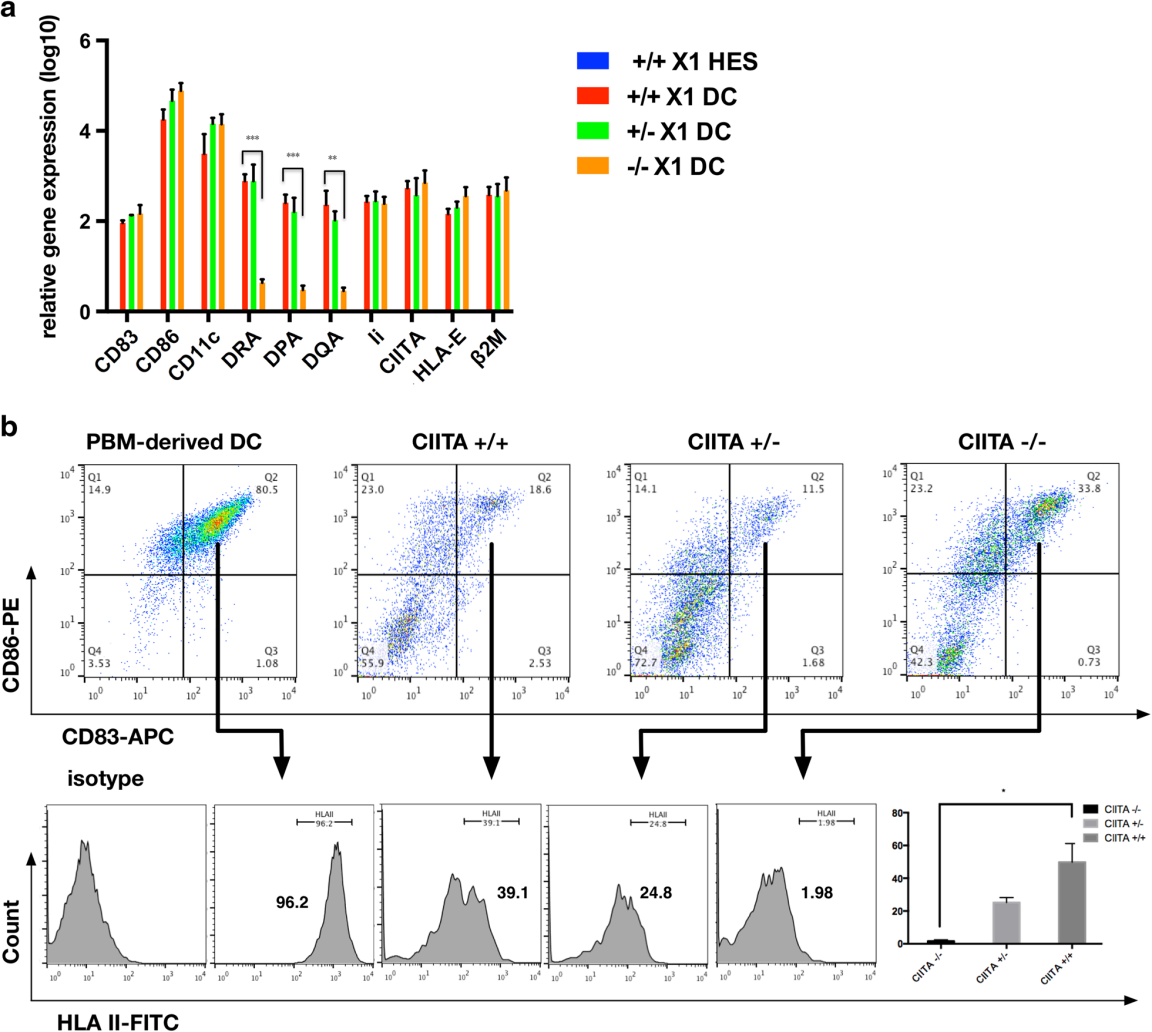
HLA Class II Expression in DCs derived from *CIITA* targeted hESCs. **a** RT-PCR analysis of CD83, CD86, CD11c*, DRA, DPA, DQA, Ii, CIITA, HLA*-*E* and *β2M* in DCs derived from *CIITA*-targeted hESCs. All groups were compared with *CIITA* ^+/+^ hESCs group. **b** FACS analysis of HLA II expression in DCs, which were defined by the co-expression of CD83 and CD86. And compare the percentage of HLA II^+^. Significance was assessed by a t test. The data are expressed as the mean ± SEM, n ≥3. ***P < 0.001, ** P < 0.01, * P < 0.05

Secondly, we derived DCs from hESCs to test constitutive HLA II expression. Focused on clinical using, we chose a protocol with definitive chemical composition media without serum, feeder or other animal products [25]. DCs derived from hESCs express CD83 and CD86 [25] (Fig. 4a, b). Compared with *CIITA*^+/+^ and *CIITA*^+/−^ DCs, lower level of classical HLA II molecules (*DRA, DQA* and *DPA*) mRNA expression was found in *CIITA*^−/−^ DCs dramatically (Fig. 4a). However, non-classical HLA II genes (*Ii*) did not show any difference in mRNA expression among them (Fig. 4a). Both classical HLA II genes (HLA-DP, HLA-DQ and HLA-DR) and non-classical HLA II genes (HLA-DM, HLA-DO, *Ii*) have a same specific regulatory module, which can be recognized by RFX-*CIITA* complex. Previous researches had shown that only HLA-DR expression was completely dependent on *CIITA*, which may cause the residual expression of other HLA II molecules in *CIITA*-targeted cells (Figs. 3a, 4a) [26, 27]. Obviously, *Ii* had different trends between DCs and fibroblasts, and it indicated a different regulatory pathway of *Ii* independent of *CIITA*. The expression of *Ii* in IFN-γ induced fibroblasts and DCs may both depend mainly on *CIITA*, while DCs differentiation last such a long time to activate the substituted regulation pathway without *CIITA*. Fortunately *Ii* is encoding accessory proteins, which is required for peptide loading of HLA II molecules [16] and can’t rescue the loss of *DRA, DPA* and *DQA* on the cell surface (Figs. 3d, 4b).

We defined DCs with CD83 and CD86, and compared the percentage of HLA II^+^ cells in CD83^+^CD86^+^ DCs. PBM derived DCs showed high overlap of those three markers (Fig. 4b). *CIITA*^−/−^ DCs only had 1.98 % HLA II^+^ cells while *CIITA*^+/+^ and *CIITA*^+/−^ DCs had higher percentage of HLA II^+^ cells, 39.1 and 24.8 % respectively.

## Conclusion

We established HLA II-deficient hESCs by disrupting *CIITA* successfully. Fibroblasts and DCs derived from those gene-modified hESCs showed defect in induced and constitutive expression of HLA II molecules, respectively. It indicated that cells derived from *CIITA*^−/−^ hESCs could escape from the attack of CD4^+^ T cells when transplanted into human body, because they were HLA II free. We combined *β2M* and *CIITA* deletion in hESCs and further transplant experiments should be done to test the affect of immune escape in humanized mice.

## Methods

### hESCs culture

hESCs were cultured and passaged as our previous paper described [11]. In brief, hESCs were cultured with the Irradiated CF1 feeder cells (3×10^4^ cells/cm^2^) on the T25 flasks (Corning) coated with Matrigel (Becton–Dickinson). hESCs were maintained in DMEM/F12 (Invitrogen) supplemented with 20 % knockout serum replacement (Invitrogen), 4 ng/mL basic fibroblast growth factor (bFGF; Invitrogen), 2 mmol/L l-glutamine (Invitrogen), 1 % nonessential amino acids (Invitrogen) and 0.1 mmol/L β-mercaptoethanol (Sigma-Aldrich). hESCs were passaged approximately once a week. Collagenase IV was used to dissociate the cells from the feeders as cell clumps, which were dissociated to an appropriate size before being passaged onto newly prepared feeder cells.

### TALENs efficiency detection

TALENs for *CIITA* were designed to target exon2 (2L1: gctgaccccctgtgcct; 2L2: gaccccctgtgcctct; 2R1: ctccagccaggtccatct; 2R2: tctccagccaggtccat) and exon3 (3L1: tcagcaggctgttgt; 3L2: tcagcaggctgttgtgt; 3R1: ccctggtctcttcat; 3R2: aagcctccctggtctt; 3R3: aagcctccctggtct). The TALENs were constructed with FastTALE TALEN Assembly Kit (Sidansai), and their activities were confirmed in 293T cells as previous description [11]. The constructed TALENs were transfected into 293T cells and selected with 2 μg/ml puromycin (Sigma). The genomic DNA of 293T cells was harvested after selection. Then, PCR and sequencing were performed to examine the efficiency of the TALENs.

### Generation of *CIITA*-deficient hESCs

To prepare the cells for transfection, harvested hESCs were plated in six well plates coated with Matrigel in mTeSR™1 medium (Stemcell Technologies). On the following day the most efficient TALENs (2L2 and 2R2) plasmids and EGFP-Puro plasmid (Sidansai) (1:1:1) were transfected into hESCs by the FuGENE HD transfection reagent (Promega). We incubated the FuGENE HD Transfection Reagent/plasmids/Opti-MEM (Life Technologies) mixture (15 ul/6 ug/300 ul) for 15 min at room temperature, and then the mixture was added into the cell culture. Puromycin was added into media two days later. After selection with 0.5 μg/ml puromycin the survival colonies were dissociated into single cells using TrypLE (Invitrogen) and seeded onto CF1-coated plates at a density of 500 cells/cm^2^. Two weeks after passaging, the colonies derived from the single cells were transferred into freshly CF1-coated wells, and in parallel, a direct cell PCR kit was used to identify the mutants.

### Teratomas formation and derivation of human fibroblasts from teratomas

hESCs were injected intramuscularly into 6–8 weeks NOD/SCID mice (approximately 5 × 10^6^ cells per site). After about 2 months, the tumors were processed for hematoxylin-eosin (HE) staining.

The fibroblast-like cells were also derived from teratomas [28]. Teratomas were cut into pieces with scissors and cultured in DMEM supplemented with 10 % serum, 1 % Pen-Strep, and 50 uM β-mecaptoethanol. After several passages, the adherent cells become homogenous and fibroblast-like cells. Cell morphological observation and RT-PCR were performed (Additional file 2: Figure S1a, b). Ten cell lines were established (3 for +/+; 3 for +/–; 4 for −/−). And we analyzed some mesenchymal stem cells markers in established cells lines (n > 3) (Additional file 2: Figure S1c). CCD and mesenchymal stem cells (MSC) were used as control. Those cell lines were more like fibroblasts. It showed that this method was reproducible in our experiments. All of the animal experiments were conducted in accordance with the Guide for the Care and Use of Animals for Research Purposes and approved by the Zhejiang University Animal Care Committee.

### Reverse Transcription-PCR and Real-Time PCR analysis

Total RNA was prepared using an RNeasy kit (Qiagen), which was then used as a template for real-time PCR (RT-PCR). RT-PCR was performed in an Eppendorf Mastercycler^®^ ep realplex real-time PCR system using SYBR Green-based PCR Master mix (TOYOBO). Standard curves were acquired for both the genes of interest and internal control (G3PD). The primers used are listed in Additional file 3: Table 1.

### Immunochemistry

Immunochemistry was performed as previously described [29]. The primary antibodies used were anti-Oct4 (1:100, Santa Cruz Biotechnology), anti-Nanog (1:150, Santa Cruz Biotechnology), anti-Tra-1-60 (1:150, Chemicon), anti-SSEA3 (Ascites, 1:400, Developmental Studies Hybridoma Bank), anti-*CIITA* (1:200, Santa Cruz Biotechnology) and anti-HLA DR+DP+DQ (1:200, Abcam) for staining.

### Western blotting

Protein extracts were obtained by lysing cells. Samples were fractionated by SDS-PAGE and transferred to polyvinylidene-fluoride (PVDC) membrane. After blocking with 5 % milk in PBST (phosphate-buffered saline with 0.1 % tween 20) for 1 h at room temperature, the membranes were probed with the corresponding primary and secondary antibodies. And the following antibodies were used: anti-HLA DR+DP+DQ (1:200, Abcam), anti-*CIITA* (1:200, Santa Cruz Biotechnology) and anti-*β*-*Actin* (1:5000, HuanAn).

### Derivation of human DCs from hESCs

We induced the differentiation of DCs from hESCs by step-wise growth factors induction in suspension culture of EBs as previously reported [25]. In the first 5 days, hESCs showed mesoderm specification in the X-VIVO™ 15 medium (Lonza) supplemented with 1 mM sodium pyruvate, 1× non-essential amino acids, 2 mM l-glutamine, 50 mM 2-mercaptoethanol and the four growth factors, including recombinant human bone morphogenetic protein-4 (rhBMP-4; BD), recombinant human vascular endothelial growth factor (rhVEGF; R&D), recombinant human granulocyte-macrophage colony-stimulating factor (rhGM-CSF; R&D) and recombinant human stem cell factor (rhSCF; R&D). From day 6 to day 10, rhBMP-4 was removed, and the cells became hematopoietic stem cells (HSCs). From day 11 to day15, rhVEGF was removed and HSCs turned into common myeloid progenitors (CMP). From day 16 to day 20, rhSCF was removed and monocyte-like cells appeared and accumulated gradually as DC precursors. DC precursors will become immature DCs (iDCs) with the treatment of rhGM-CSF and recombinant human Interleukin 4 (rhIL-4; R&D) in the next 4–6 days. The maturation of DCs needed further incubation for 1–2 days with the mixed factors, including rhGM-CSF, recombinant human Interleukin-1 beta (rhIL1-β; R&D), recombinant human Interferon gamma (rhIFN-γ; R&D), Prostaglandin E2 (PGE-2; Sigma) and recombinant human Tumor necrosis factor alpha (rhTNF-α; R&D).

### Fluorescence-activated cell sorting

Cells were dissociated with TrypLE and were stained for 30 min at 4 °C in 100 μL of 0.5 % FBS in PBS containing an appropriate dilution of PE, FITC, APC or PE-CY™7-conjugated antibody. Primary antibodies included human CD83, CD86, HLA II and HLA I (BD Biosciences). The sample measurement was performed on a BD FACSCalibur flow cytometer system, and the analysis was performed using FlowJo software (Tree Star, Ashland, OR).

### Statistical analysis

All of the data are represented as the mean ± SEM. The data were analyzed statistically using GraphPad Prism 5.1 (GraphPad Software Inc., USA). All performance variants were analyzed by the unpaired Student’s *T* test. Statements of significance were based on *P < 0.05 unless otherwise stated.

## Additional files

10.1186/s40659-015-0051-6 Potential Talen off-targets analysis.
10.1186/s40659-015-0051-6 Derivation of Human Fibroblasts from Teratomas. (A) The morphology of fibroblasts derived from hESCs. (B) Fibroblast derived from hESCs express Vimentin. All groups were compared with *CIITA* ^+/+^ fibroblasts group. (C) Fibroblast derived from hESCs compare with MSC. MSC markers (CD73, CD90, CD105, CD44) were checked.
10.1186/s40659-015-0051-6 List of RT-PCR primers.

## References

[1] Thomson JA, Itskovitz-Eldor J, Shapiro SS, Waknitz MA, Swiergiel JJ, Marshall VS (1998). Embryonic stem cell lines derived from human blastocysts. Science.

[2] Smith-Garvin JE, Koretzky GA, Jordan MS (2009). T cell activation. Annu Rev Immunol..

[3] Fife BT, Bluestone JA (2008). Control of peripheral T-cell tolerance and autoimmunity via the CTLA-4 and PD-1 pathways. Immunol Rev.

[4] Rong ZL, Wang MY, Hu Z, Stradner M, Zhu SY, Kong HJ (1998). An effective approach to prevent immune rejection of human esc-derived allografts. Cell Stem Cell.

[5] Porter DL, Levine BL, Kalos M, Bagg A, June CH (2011). Chimeric antigen receptor-modified T cells in chronic lymphoid leukemia. N Engl J Med.

[6] Wei HF, Wang H, Lu B, Li BH, Hou S, Qian WZ (2008). Cancer immunotherapy using in vitro genetically modified targeted dendritic cells. Cancer Res.

[7] Senju S, Hirata S, Motomura Y, Fukuma D, Matsunaga Y, Fukushima S (2010). Pluripotent stem cells as source of dendritic cells for immune therapy. Int J Hematol.

[8] Landais E, Romagnoli PA, Corper AL, Shires J, Altman JD, Wilson IA (2009). New design of MHC class II tetramers to accommodate fundamental principles of antigen presentation. J Immunol..

[9] Zimmer J, Andres E, Donato L, Hanau D, Hentges F, de la Salle H (2005). Clinical and immunological aspects of HLA class I deficiency. s.

[10] Rodriguez T, Mendez R, Del Campo A, Aptsiauri N, Martin J, Orozco G (2007). Patterns of constitutive and IFN-gamma inducible expression of HLA class II molecules in human melanoma cell lines. Immunogenetics.

[11] Lu P, Chen J, He L, Ren J, Chen H, Rao L (2013). Generating hypoimmunogenic human embryonic stem cells by the disruption of beta 2-microglobulin. Stem Cell Rev..

[12] Riolobos L, Hirata RK, Turtle CJ, Wang PR, Gornalusse GG, Zavajlevski M (2013). HLA engineering of human pluripotent stem cells. Mol Ther.

[13] Haruta M, Tomita Y, Yuno A, Matsumura K, Ikeda T, Takamatsu K (2013). TAP-deficient human iPS cell-derived myeloid cell lines as unlimited cell source for dendritic cell-like antigen-presenting cells. Gene Ther.

[14] Mach B, Steimle V, Reith W (1994). MHC class II-deficient combined immunodeficiency: a disease of gene regulation. Immunol Rev.

[15] Reith W, LeibundGut-Landmann S, Waldburger JM (2005). Regulation of MHC class II gene expression by the class II transactivator. Nat Rev Immunol.

[16] Reith W, Mach B (2001). The bare lymphocyte syndrome and the regulation of MHC expression. Annu Rev Immunol.

[17] van den Elsen PJ, van der Stoep N (2003). Class II transactivator (CIITA) deficiency in tumor cells: complicated mechanisms or not?. Am J Pathol..

[18] Odeberg J, Plachter B, Branden L, Soderberg-Naucler C (2003). Human cytomegalovirus protein pp65 mediates accumulation of HLA-DR in lysosomes and destruction of the HLA-DR alpha-chain. Blood.

[19] Stumptner-Cuvelette P, Morchoisne S, Dugast M, Le Gall S, Raposo G, Schwartz O (2001). HIV-1 Nef impairs MHC class II antigen presentation and surface expression. Proc Natl Acad Sci U S A..

[20] Chang CH, Guerder S, Hong SC, van Ewijk W, Flavell RA (1996). Mice lacking the MHC class II transactivator (CIITA) show tissue-specific impairment of MHC class II expression. Immunity.

[21] Drukker M, Katz G, Urbach A, Schuldiner M, Markel G, Itskovitz-Eldor J (2002). Characterization of the expression of MHC proteins in human embryonic stem cells. Proc Natl Acad Sci USA..

[22] Gaj T, Gersbach CA, Barbas CF (2013). ZFN, TALEN, and CRISPR/Cas-based methods for genome engineering. Trends Biotechnol.

[23] Wu Z, Li H, Rao L, He L, Bao L, Liao J (2011). Derivation and characterization of human embryonic stem cell lines from the Chinese population.. J Genet Genomics.

[24] Doyle EL, Booher NJ, Standage DS, Voytas DF, Brendel VP, Vandyk JK (2012). TAL Effector-Nucleotide Targeter (TALE-NT) 2.0: tools for TAL effector design and target prediction. Nucleic Acids Res..

[25] Silk KM, Tseng SY, Nishimoto KP, Lebkowski J, Reddy A, Fairchild PJ (2011). Differentiation of dendritic cells from human embryonic stem cells. Methods Mol Biol.

[26] Hake SB, Tobin HM, Steimle V, Denzin LK (2003). Comparison of the transcriptional regulation of classical and non-classical MHC class II genes. Eur J Immunol.

[27] Tai AKF, Zhou G, Chau K, Ono SJ (1999). Cis-element dependence and occupancy of the human invariant chain promoter in CIITA-dependent and -independent transcription. Mol Immunol.

[28] Rong Z, Fu X, Wang M, Xu Y (2012). A scalable approach to prevent teratoma formation of human embryonic stem cells. J Biol Chem.

[29] Xiao L, Yuan X, Sharkis SJ (2006). Activin A maintains self-renewal and regulates fibroblast growth factor, Wnt, and bone morphogenic protein pathways in human embryonic stem cells. Stem Cells.

