# Supplementary figures and images for: Functional disruption of human leukocyte antigen II in human embryonic stem cell
Source: Biol Res. 2015 Oct 27;48:59. doi: 10.1186/s40659-015-0051-6 (PMC4624597; doi:10.1186/s40659-015-0051-6)

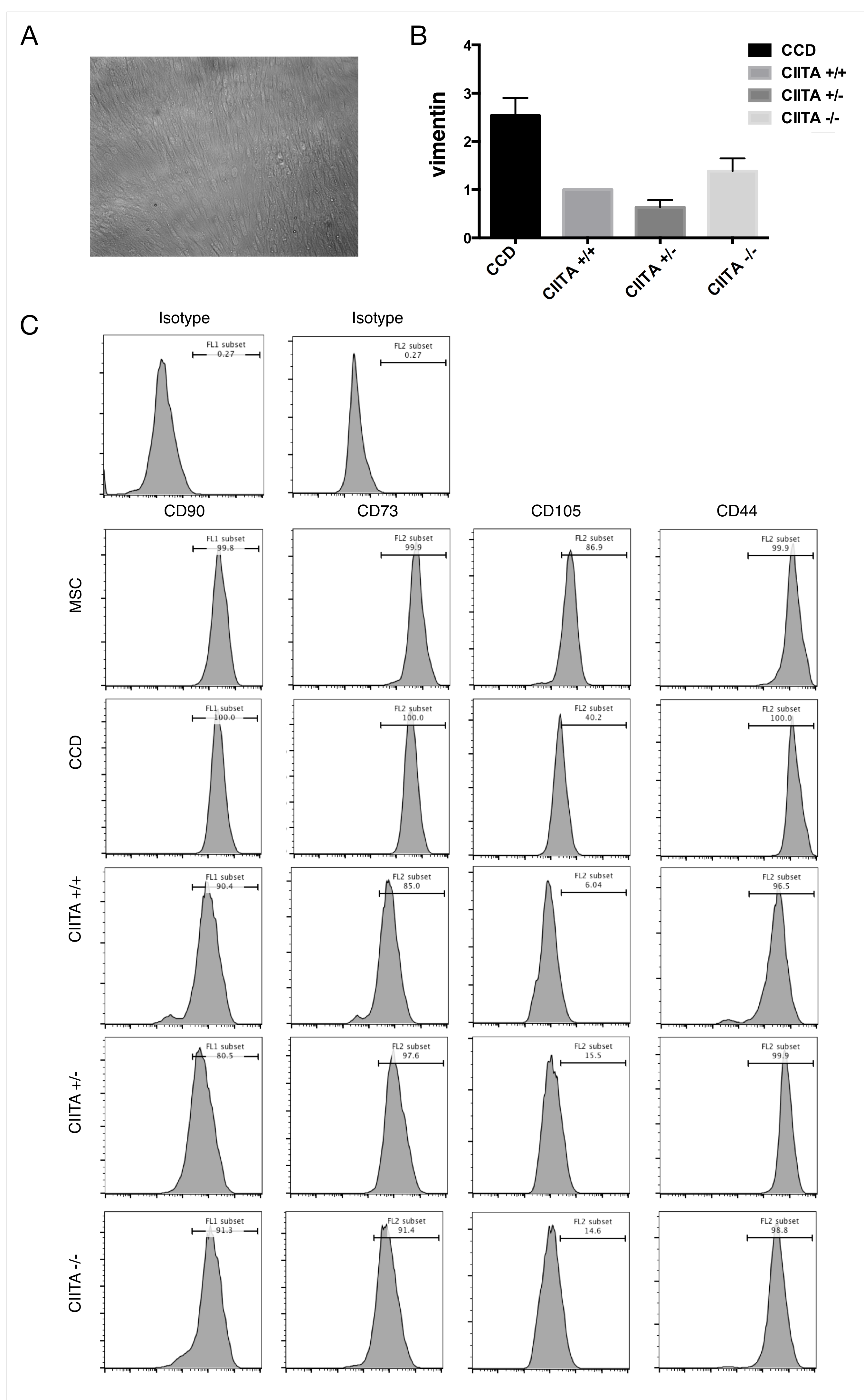

Supplement: Supplementary file 2 — 10.1186/s40659-015-0051-6 Derivation of Human Fibroblasts from Teratomas. (A) The morphology of fibroblasts derived from hESCs. (B) Fibroblast derived from hESCs express Vimentin. All groups were compared with CIITA +/+ fibroblasts group. (C) Fibroblast derived from hESCs compare with MSC. MSC markers (CD73, CD90, CD105, CD44) were checked. [file 40659_2015_51_MOESM2_ESM.tiff]
